# Social Support and Mental Health of Older Adults in Sub-Saharan Africa: An Exploratory Chain Mediation Analysis

**DOI:** 10.1093/geroni/igaf122.4200

**Published:** 2025-12-31

**Authors:** Wisdom Obioha, Nkechi Chukwuemeka, Ikenna Okechi, Ifeoma Eze

**Affiliations:** Miami University, Oxford, Ohio, United States; University of Nigeria, University of Nigeria, Enugu, Nigeria; University of Nigeria, University of Nigeria, Enugu, Nigeria; University of Nigeria, University of Nigeria, Enugu, Nigeria

## Abstract

Social support has regularly been identified as both protective and promotive factor for the mental health of older adults in various contexts and cultures, yet these benefits of social support may require other important factors to be transmitted to the mental health of older adults, especially in Sub-Saharan Africa. Also, given that social support is a multidimensional construct, different types of social support may have varying levels of benefits for older adults, especially when interacting with other factors. We employed survey data from 350 older adults aged between 60 and 93 years old from a rural community in Nigeria to explore the potential sequential mediation effect of self-compassion and life satisfaction on older adults’ mental health. Data was derived from the Multidimensional Scale of Perceived Social Support, Self-Compassion Scale-Short Form, Satisfaction with Life Scale, and Psychological Well-being Scale. After controlling for age, marital status, and education; family support, self-compassion and life satisfaction were positive predictors of mental health. Life satisfaction was a significant mediator between social support and mental health, but self-compassion was not. Support from friends and significant others associated positively with mental health only through the mediating effect of life satisfaction. Self-compassion and life satisfaction also did not exert sequential mediation effects on this relationship. The study concludes that self-compassionate older adults who perceive a sufficient amount of support from important sources and maintain high levels of life satisfaction may witness significant improvements in their mental health. Concerted efforts should be tailored towards improving self-compassion among older adults.

